# Prevalence and associated factors for alcohol use disorder among tuberculosis patients: a systematic review and meta-analysis study

**DOI:** 10.1186/s13011-020-00335-w

**Published:** 2021-01-03

**Authors:** Mogesie Necho, Mekonnen Tsehay, Muhammed Seid, Yosef Zenebe, Asmare Belete, Habitam Gelaye, Amare Muche

**Affiliations:** 1 College of Medicine and Health Sciences, Department of Psychiatry, Wollo University, Dessie, Ethiopia; 2 College of Medicine and Health Sciences, Department of Public Health, Wollo University, Dessie, Ethiopia

**Keywords:** Alcohol use disorder, Tuberculosis, Africa, Systematic review, Meta-analysis

## Abstract

**Background:**

Alcohol use disorders (AUD) in tuberculosis patients are complicated with poor compliance to anti-tuberculosis treatment and poor tuberculosis treatment outcomes. However, aggregate data concerning this problem is not available. Therefore, this review aimed to fill the above gap by generating an average prevalence of AUD in tuberculosis patients.

**Method:**

Our electronic search for original articles was conducted in the databases of Scopus, PubMed, and EMBASE, African Index Medicus, and psych-info. Besides, the reference list of selected articles was looked at manually to have further eligible articles for the prevalence and associated factors of AUD in tuberculosis patients. The random-effects model was employed during the analysis. MS-Excel was used to extract data and stata-11 to determine the average prevalence of AUD among tuberculosis patients. A sub-group analysis and sensitivity analysis were also run. A visual inspection of the funnel plots and an Eggers publication bias plot test were checked for the presence of publication bias.

**Result:**

A search of the electronic and manual system resulted in 1970 articles. After removing duplicates and unoriginal articles, only 28 articles that studied 30,854 tuberculosis patients met the inclusion criteria. The average estimated prevalence of AUD in tuberculosis patients was 30% (95% CI: 24.00, 35.00). This was with a slight heterogeneity (I^2^ = 57%, *p*-value < 0.001). The prevalence of AUD in tuberculosis patients was higher in Asia and Europe; 37% than the prevalence in the US and Africa; 24%. Besides, the average prevalence of AUD was 39, 30, 30, and 20% in studies with case-control, cohort, cross-sectional and experimental in design respectively. Also, the prevalence of AUD was higher in studies with the assessment tool not reported (36%) than studies assessed with AUDIT. AUD was also relatively higher in studies with a mean age of ≥40 years (42%) than studies with a mean age < 40 years (24%) and mean age not reported (27%). Based on a qualitative review; the male gender, older age, being single, unemployment, low level of education and income from socio-demographic variables, retreatment and treatment failure patients, stigma, and medication non-adherence from clinical variables were among the associated factors for AUD.

**Conclusion:**

This review obtained a high average prevalence of AUD in tuberculosis patients and this varies across continents, design of studies, mean age of the participants, and assessment tool used. This implied the need for early screening and management of AUD in tuberculosis patients.

**Supplementary Information:**

The online version contains supplementary material available at 10.1186/s13011-020-00335-w.

## Background

Tuberculosis (TB) [1] is a major public health problem in the world. TB is caused by bacteria (*Mycobacterium tuberculosis*) [2] and it most often affects the lungs. TB is spread through the air when people with lung TB cough, sneeze or spit. A person needs to inhale only a few germs to become infected. Despite being a preventable and curable disease, it the world’s top infectious killer that 1.5 million people die from TB each year [3]. Although there are numerous global efforts to control tuberculosis (TB), it remains a chronic infectious disease with high morbidity and mortality in several parts of the world [3–5].

Several studies carried out in the world have shown alcohol use disorder as a risk factor for tuberculosis mortality, factor for default in TB, and reason for non-compliance [6]. Alcohol is a toxic and psychoactive substance. Diagnostic and statistical manual of mental disorders, 5th edition, defines Alcohol use disorder as a problematic pattern of alcohol use leading to clinically significant impairment or distress as manifested by at least 2 symptoms criteria over the same 12-month period [7]. Based on ICD 10 criteria Alcohol use disorders is for alcohol dependence and harmful use (F10.1 and F 10.2), excluding cases with a comorbid depressive episode [8].

Alcohol consumption contributes to 3 million deaths each year globally as well as the leading risk factor for premature mortality and disability among those aged 15 to 49 years. Overall, the harmful use of alcohol is responsible for 5.1% of the global burden of disease [9, 10].

There are different rates of prevalence of alcohol use disorder among TB patients across developed and developing countries. For example, a study in the United States reported that the 1-year prevalence of AUD in tuberculosis patients was 24.7% [11]. Similarly, in India, the 1-year prevalence of AUD among TB patients was 29% [12]. In Africa too, the 1-year prevalence of alcohol use disorder among tuberculosis patients was found to be 34.7% in Zambia [13], 23.2% in South Africa [14], and 35.1% in Botswana [15].

There have been numerous publications describing, the impact of alcohol use disorders among TB patients [12, 16–18]. Studies show, the risk of active tuberculosis, re-infection of TB, and TB treatment non-adherence is substantially increased in people who have an alcohol use disorder. The possible reason commonly reported was an influence on the immune system of alcohol itself and of alcohol-related conditions [16, 19, 20].

Alcohol use disorder has also may result in an increased chance of liver damage among TB patients and alter the metabolism of antibacterial drugs [21]. In a review done in Russia, alcohol consumption during treatment was a significant predictor of poor treatment outcomes which lead to MDR-TB [22]. Alcohol use disorders influence not only the incidence of tuberculosis but also its clinical evolution and outcome, a meta-analysis review on the impact of alcohol use on tuberculosis treatment outcomes, show it increased the risk of poor treatment outcomes in both drug-susceptible and MDR-TB patients [23].

The most commonly reported associated factors of alcohol use in TB patients include, male gender older age, Poor perceived health status, tobacco use, psychological distress, being a TB retreatment patient, among women lower education, and tobacco use [12, 14, 24, 25].

Even though a wide range of studies showed AUD as significant public health importance, there is no systematic review and meta-analysis conducted to assess the prevalence of AUD among TB patients. Therefore, this systematic review and meta-analysis aimed to summarize the existing evidence on the prevalence of AUD among TB patients and to formulate possible suggestions for future clinical practice and research community.

## Methods

### Search process

We conducted this systematic review and meta-analysis on studies that examined alcohol use disorder and associated factors in tuberculosis patients who are on anti-tuberculosis treatment. In doing this research, the preferred reporting items for systematic reviews and meta-analyses guideline [26] have been followed. A comprehensive search of available literature was done in the databases of Embase, Scopus, PubMed, Psych-info, and African Index Medicus to recruit original research articles published between September 2007 and October 2020 (Supplementary file 1). Non-indexed articles from Google scholar, WHO websites, other institutional repositories, and manually searched reference lists of included studies were also part of the review.

### Eligibility criteria

Original quantitative studies that examined the alcohol use disorder and associated factors in tuberculosis patients on anti-tuberculosis treatment were included. The studies included were randomized controlled trials, cohort, case-control, and cross-sectional in design. Studies were not eligible for inclusion if they: 1) Published in a language other than English; 2) were conducted in non-human subjects 3) did not assess alcohol use disorder in tuberculosis patients with a validated assessment instrument; 4) were not concerned with the exposure (tuberculosis) and outcome (alcohol use disorder) of the review. Two of the review authors (M.T and A.B) independently conducted the search process. A three-stage screening of the searched data was performed. At the initial stage, the authors screened the titles of the articles. In the second stage, the abstract of articles included in the first stage was done. In the final stage, the full paper of an article was done to assess the eligible articles for inclusion. If two of the above-mentioned researchers had a different point of view on whether or not to include an article, the senior research author (M.N.) was referred to make the final judgment.

### Data extraction and appraisal of study quality

We extracted data on Microsoft-excel from the included studies using a standard data extraction template. The template consisted of the author, publication year, population and phases of treatment (Tuberculosis or MDR-TB patients at DOT/continuation phase of treatment), socio-demographic characteristics (review population, sex, and age), region, the tool used, and prevalence of alcohol use disorder. The quality of studies included in the final analysis was evaluated with the Johanna Briggs Institute (JBI) quality assessment checklist [27–29]. The components of the JBI quality assessment checklist includes; appropriateness in the description of review subjects and setting, adequacy of the sample size, the appropriateness of sample frame, sampling procedure of participants, appropriateness of data analysis, usage of valid measurement, and reliability of measurement, adequacy of the response rate, adequate follow up time, complete follow-up, appropriate strategies to address lose to follow up and the use of appropriate statistical methods.

### Statistical analysis

The pooled estimated prevalence of alcohol use disorder in tuberculosis patients was done with the Stata-11 Meta-prop package [30]. Besides, all statistical operations including funnel and forest plots were done using the stata-11 software and random effect model. We employed the Higgs *I*^2^ statistics [31] to identify the presence of potential heterogeneity between the included studies. A Higgs *I*^2^ value of 50% and above during the analysis was interpreted as a significant heterogeneity [31]. As heterogeneity was a main problem of the present review, a sub-group analysis was done to detect the source of this heterogeneity. Moreover, a single review leaves out at a time sensitivity analysis was also done to identify a single review that out weighted the overall result. Eyeball test [32] and the Eggers test for publication bias were implemented to identify the existence of a small review effect. All statistical values with a *P*-value < 0.05 were interpreted as a significant value.

## Results

### Search result

**Fig. 1:**
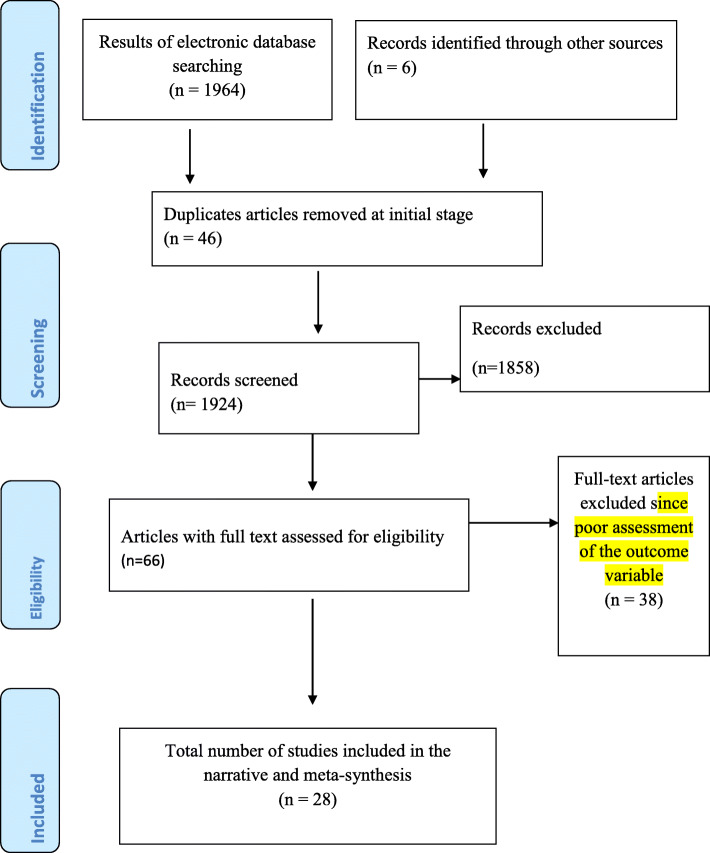
PRISMA flow chart for the search and refining steps of the study

Our electronic and manual search for eligible articles resulted in the identification of 1970 articles. From these records, 46 articles were duplicate articles and therefore removed in the initial stage. From the remaining 1924 articles, only 66 were obtained eligible for a full-text revision after the remaining were excluded at the different steps of screening. In the end, only 28 research articles were found to be eligible and included in the analysis (Fig. 1).

### Characteristics of studies included

A total of 28 studies [11–13, 15, 17, 25, 33–54] that studied our outcome of interest; alcohol use disorder (AUD) in thirty thousand eight hundred fifty-four (30854) tuberculosis patients on treatment with anti-tuberculosis medications were included in the present review. Considering the regional setting where the included studies were done; six [17, 25, 35, 37, 38, 47], five [40, 45, 46, 48, 49] and another five studies were from Russia, South Africa [33, 34, 50, 51, 53] and Ethiopia [33, 34, 50, 51, 53] respectively. The remaining studies were from United States (US) [11, 36], Estonia [41, 42], India [12, 43, 52, 54], Thailand [44], Nigeria [39], Botswana [15], and Zambia [13]. Most of the studies in the present analysis were Cohort [25, 35, 38, 40, 44, 46, 48, 51–53] and cross-sectional [12, 13, 17, 33, 34, 36, 37, 41, 43, 45, 49, 50].

One -third of the studies included [11, 12, 15, 17, 25, 34, 44–49, 51–53] used the Alcohol use disorder identification test (AUDIT) to measure alcohol use disorder in tuberculosis patients. Besides two studies [13, 39] measured AUD with mini-international neuropsychiatric-interview(MINI), one [33] with alcohol, smoking, and substance involvement screening test(ASSIST), and another one used DSM-IV [37]. However, eight of the studies [35, 36, 38, 40–43, 50] did not report the assessment tool for the measurement of AUD. Regarding the setting of anti-tuberculosis treatment, seven [13, 17, 33, 45, 46, 48, 49], 13 [11, 12, 25, 35, 38–41, 43, 44, 47, 52] and another seven [34, 36, 42, 50, 51, 53] of the included studies involved subjects with treatment setting at the primary health care setting (PHCU), hospital and both hospital and PHCU respectively. Also, 20 [11, 12, 33, 35, 37, 38, 43–46, 49, 51–53], four [17, 25, 42, 47] and three [34, 36, 50] of the studies involved participant patients at the directly observed treatment(DOT), continuation and both phases of ant-tuberculosis treatment in the respective order (Table 1).

**Table 1:** Characteristics of studies included on the meta-analysis of Alcohol use disorder in tuberculosis patients

Author	Year	Region	Design	Setting	Study population	Tool	Mean/media age(year)	Phase of Rt	AUD by sex/n (%)		Outcome		
									Male n(%)	Female n(%)	AUD n(%)	Abuse n(%)	Dependence n(%)
Fiske et al. [36]	2009	US	CS	All settings	5556	NA	NA	All phases	1130 (30.6)	196 (10.5)	1326(23.8)	NA	NA
Hayes-larson et al. [11]	2017	US	RCT	Hospital	371	AUDIT	35	DOT phase	NA	NA	(24.7)	NA	NA
Fleming et al. [37]	2006	Russia	CS	Hospital	200	DSM-IV	41	DOT phase	NA	NA	125 (62.5)	40(20)	85(42.5)
Mathew et al. [55]	2009	Russia	CS	PHCU	851	AUDIT	NA	Continuation	NA	NA	469 (55.1)	NA	NA
Miller et al. [47]	2016	Russia	RCT	Hospital	196	AUDIT	NA	Continuation	NA	NA	22(11.2)	NA	NA
Shin et al. [25]	2010	Russia	Cohort	Hospital	374	AUDIT	41.1	Continuation	112(39.7)	16(17.4)	128(57.1)	45(21.1)	83(36.0)
Gelmanova et al. [38]	2007	Russia	Cohort	Hospital	237	NA	40	DOT phase	NA	NA	57(24)	NA	NA
Cavanaugh et al. [35]	2012	Russia	Cohort	Hospital	200	NA	42	DOT phase	NA	NA	103(51.5)	NA	NA
Laprawat et al. [44]	2017	Thailand	Cohort	Hospital	295	AUDIT	NA	DOT phase	NA	NA	72(24)	NA	NA
Thomas et al. [52]	2019	India	Cohort	Hospital	455	AUDIT	38	DOT phase	NA	NA	45(10)	NA	NA
Suhadev et al. [12]	2011	India	CS	Hospital	490	AUDIT	NA	DOT phase	NA	NA	63(12.8)	41(8.4)	22(4.5)
Kulkarni et al. [43]	2013	India	CS	Hospital	156	NA	33	DOT phase	NA	NA	54(34.6)	NA	NA
Thummar 2020 [54]	2020	India	CS	NA	200	AUDIT	NA	NA	NA	NA	NA	40(20%)	Hazardous drinking
Kliman et al. [56]	2010	Estonia	CS	Hospital	1163	NA	45.3	NA	NA	NA	462(39.7)	NA	NA
Kliman et al. [42]	2009	Estonia	CC	All settings	1109	NA	43.2	Completed treatment	NA	NA	469(42.3)	NA	NA
Author	Year	Region	design	Setting	Study population	Tool	Mean/media age	Phase of Rt	AUD by sex/n (%)		Outcome		
									Male	Female	AUD n (%)	Abuse	Dependence
Louw et al. [45]	2012	SA	CS	PHCU	4900	AUDIT	36.2	DOT phase	NA	NA	1142(23.3)	NA	NA
Peltzer et al. [48]	2014	SA	CS	PHCU	4900	AUDIT	36.2	DOT phase	820(31.8)	280(13)	NA	NA	NA
Kedall et al. [40]	2013	SA	Cohort	Hospital	225	NA	37.5	DOT phase	NA	NA	134(63)	NA	NA
Peltzer et al. [49]	2014	SA	Cohort	PHCU	1196	AUDIT	NA	DOT phase	NA	NA	321(26.8)	NA	NA
O,connel et al. [13]	2013	Zambia	CS	PHCU	649	MINI	NA	NA	127(32.3)	15(5.8)	142 (21.8)	25(3.8)	117(18)
Tola et al. [53]	2015	Ethiopia	Cohort	All setting	330	AUDIT	32.21	DOT phases	NA	NA	62 (18.8)	NA	NA
Ayana et al. [34]	2019	Ethiopia	CS	All setting	365	AUDIT	35.5	All phases	NA	NA	16 (4.4)	NA	NA
Tesfahugn et al. [50]	2015	Ethiopia	CS	All setting	200	NA	34.9	All phases	NA	NA	36 (18)	NA	NA
Tesfaye et al. [51]	2019	Ethiopia	Cohort	All setting	268	AUDIT	NA	DOT phases	NA	NA	29 (10.8)	NA	NA
Ambaw et al. [33]	2017	Ethiopia	CS	PHCU	657	ASSIST	30	DOT phases	NA	NA	89 (9.3)	NA	NA

Key: *AUD* Alcohol Use Disorder, *AUDIT* Alcohol Use Disorder Identification Test, *ASSIST* Alcohol Smoking and Substance Involvement Screening Test, *CC* Case control, *CS* Cross-sectional, *DSM-IV* Diagnostic and Statistical Manual of Mental Disorders, *DOT* Directly Observed Therapy, *MINI* Mini-international Neuropsychiatric Interview, *NA* Not Reported, *PHCU* Primary Health Care Unit, *RCT* Randomized Controlled Trial, *SA* South Africa, *US* United States

### The 1-year prevalence of alcohol use disorder among tuberculosis patients

**Fig. 2:**
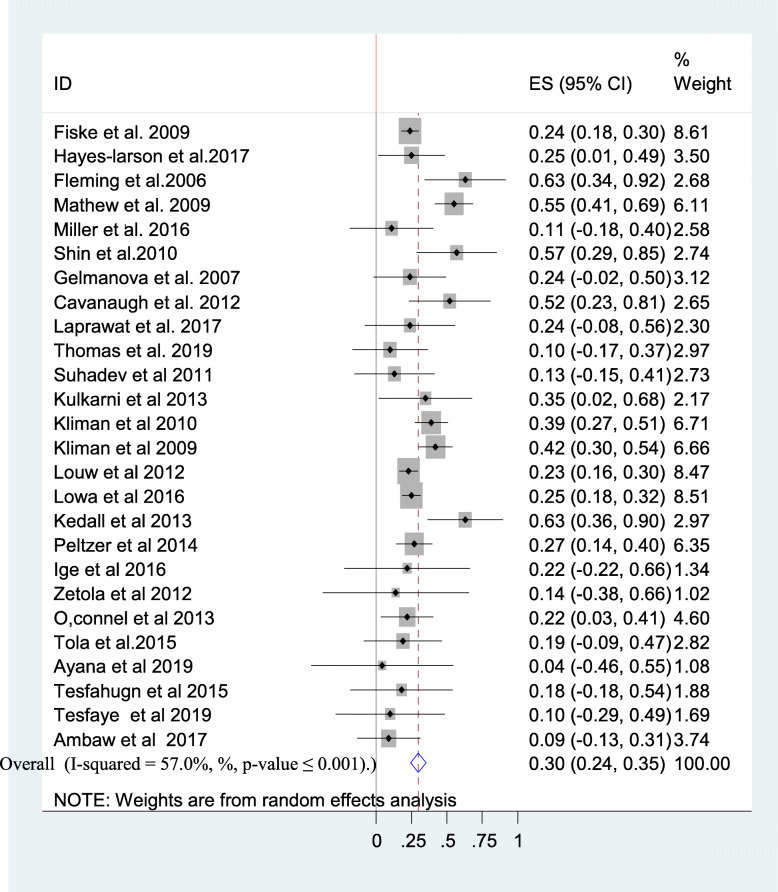
A forest plot for the prevalence of AUD

Twenty-seven studies [11–13, 15, 17, 25, 33–53] had reported the prevalence of alcohol use disorder among tuberculosis patients. The reported prevalence of alcohol use disorder among tuberculosis patients among studies included in this review ranges from 4.4% in a review from Ethiopia [34] to 63% in Russia [37] and South Africa [40]. The average prevalence of alcohol use disorder among tuberculosis patients using the random effect model was found to be 30% (95% CI: 24.00, 35.00). This average prevalence of AUD was with a slight heterogeneity (I^2^ = 57%, *p*-value < 0.001) from the difference between the 27 studies **(**Fig. 2**)**.

### The relationship between gender and AUD

Four of the included studies [13, 25, 36, 48] have reported the prevalence of AUD in line with the sex of the participants. The average prevalence of AUD in male participants as reported by the above studies was 33.6% (95% CI: 30.65, 36.55%) and this was higher than the average prevalence of AUD in females 11.67% (95% CI: 7.81, 15.54%).

### Subgroup analysis of the 1-year prevalence of alcohol use disorder among tuberculosis patients

**Fig. 3:**
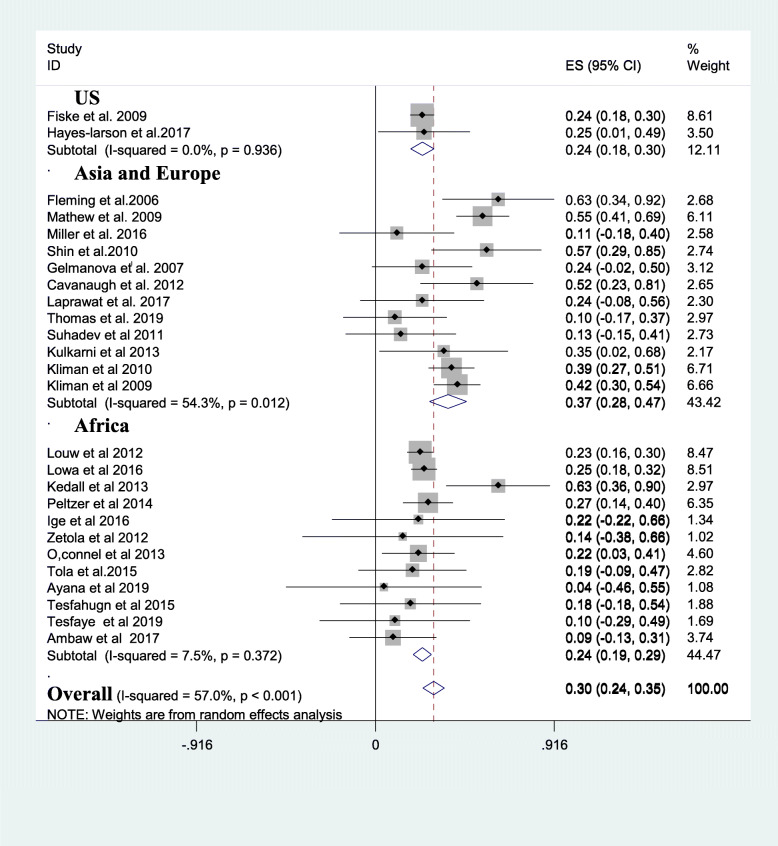
A subgroup analysis of AUD based on continent of the study. Key: AUD: Alcohol Use Disorder, US: United States of America

**Fig. 4:**
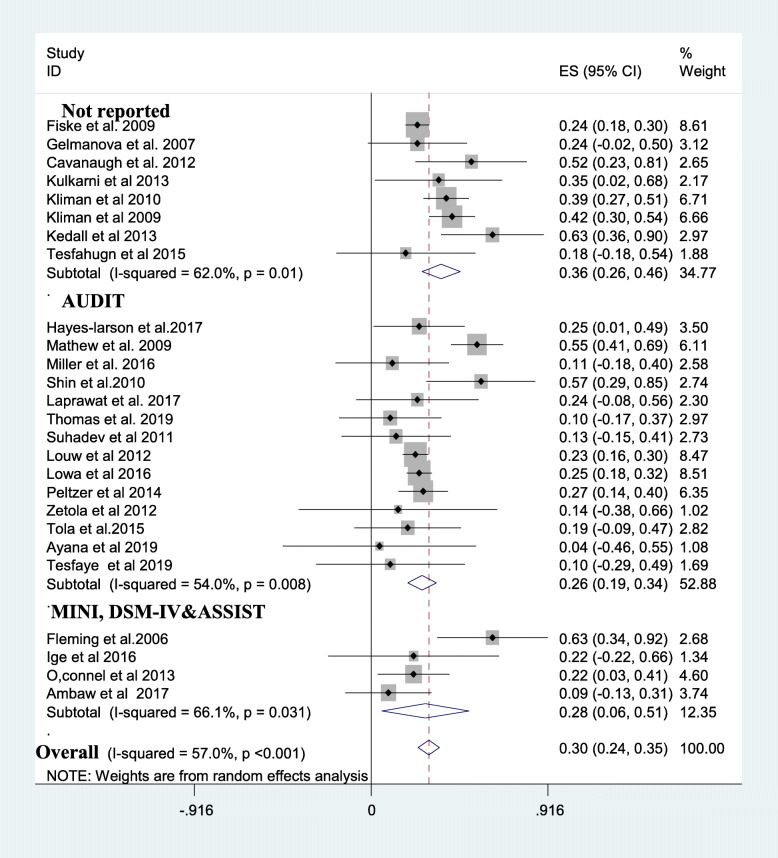
A subgroup analysis of alcohol use disorder based on measurement tools. Key: AUDIT: Alcohol Use Disorder Identification Test, ASSIST: Alcohol Smoking and Substance Involvement Screening Test,, DSM-IV: Diagnostic and Statistical Manual of Mental Disorders, MINI: Mini-international Neuropsychiatric Interview

A subgroup analysis was done considering the mean age of review participants, the continent at which the review was done, study design, and assessment tool used. The average prevalence of alcohol use disorder in tuberculosis patients was higher in Asia and Europe;37% [11, 12, 17, 25, 35–38, 41–44, 47, 52] than the prevalence in US; 24% [11, 36] and Africa; 24% [13, 15, 33, 34, 39, 40, 45, 46, 48–51, 53] **(**Fig. 3). The average prevalence of AUD was 36% in studies that do not report the assessment tool for AUD [35, 36, 38, 40–43, 50] which is higher than the prevalence in studies that utilized AUDIT (26%) [11, 12, 15, 17, 25, 34, 44–49, 51–53] **(**Fig. 4**)**. Besides, studies which were case-control [15, 39, 41] provided higher prevalence of AUD (39%) than cross-sectional [12, 13, 17, 33, 34, 36, 37, 41, 43, 45, 49, 50](30%), cohort [25, 35, 38, 40, 44, 46, 48, 51–53](30%) and RCT studies [11, 47] (20%). Last but not least the average prevalence of AUD was 42% in studies with a mean age of the participants 40 years and above higher than the average prevalence of AUD in participants with a mean age of < 40 years (24%) and mean age not reported (27%) (Table 2).

**Table 2:** A subgroup analysis of the prealence of alcohol use disorder among tuberculosis patients

Subgroup		Number of studies	Estimates		Heterogeneity	
			Prevalence	95% CI	I^2^	*P*-value
Mean age	Not reported	10	0.27	0.19, 0.34	57.3%	*P* = 0.012
	Below 40 years age	9	0.24	0.14, 0.33	35%	*P* = 0.135
	40 years and above	7	0.42	0.43, 0.50	8.2%	*P* = 0.37
Continent	US	2	0.24	0.18, 0.30	0%	*P* = 0.936
	Asia and Europe	12	0.37	0.28, 0.47	54.3%	*P* = 0.012
	Africa	12	0.24	0.19, 0.29	7.5%	*P* = 0.372
Study design	Cross-sectional	11	0.3	0.21, 0.39	70.4%	*P* ≤ 0.01
	Cohort	10	0.3	0.21, 0. 40	48%	*P* ≤ 0.01
	Case control	3	0.39	0.28, 0.51	0%	*P* = 0.43
	RCT	2	0.2	0.01, 0.38	0%	*P* = 0.47
Assessment tool	AUDIT	14	0.26	0.19, 0.34	54%	*P* = 0.008
	MINI, DSM-IV&ASSIST	4	0.28	0.08,0.51	66.1%	*P* = 0.0031
	Not reported	8	0.36	0.26, 0.46	62%	*P* = 0.01

Key: *AUDIT* Alcohol Use Disorder Identification Test, *ASSIST* Alcohol Smoking and Substance Involvement Screening Test, *CS* Cross-sectional, *DSM-IV* Diagnostic and Statistical Manual of Mental Disorders, *MINI* Mini-international Neuropsychiatric Interview, *RCT* Randomized Controlled Trial, *US* United States

### Sensitivity analysis

We further investigated the source of heterogeneity by doing a leave-one-out sensitivity analysis to identify whether individual studies out weighted the average prevalence of AUD. Our result revealed that the average prevalence of AUD obtained when each study was omitted at a time from the analysis ranges between 28% (23.00, 35.00) and 31% (25.00, 36.00). This implied that the average prevalence of AUD among tuberculosis patients was not out weighted by a single review (Table 3).

**Table 3:** A sensitivity analysis of the prevalence of alcohol use disorder among tuberculosis patients

No	Study excluded from the analysis	Average prevalence of AUD	95% confidence interval
1	Fiske et al.	0.3	0.24, 0.36
2	Hayes-larson et al	0.3	0.24, 0.36
3	Fleming et al	0.29	0.24, 0.34
4	Mathew et al	0.28	0.23, 0.33
5	Miller et al	0.3	0.25, 0.36
6	Shin et al	0.3	0.24, 0.35
7	Gelmanova et al	0.3	0.24, 0.36
8	Cavanaugh et al	0.29	0.24, 0.35
9	Laprawat et al	0.28	0.25, 0.31
10	Thomas et al	0.3	0.24, 035
11	Suhadev et al.	0.3	0.25, 0.36
12	Kulkarni et al.	0.3	0.24, 035
13	Kliman et al.	0.29	0.24, 035
14	Kliman et al.	0.29	0.23, 0.35
15	Louw et al	0.3	0.24, 0.36
16	Lowa et al	0.3	0.24, 0.36
17	Kedall et al	0.28	0.25, 0.31
18	Peltzer et al	0.3	0.24, 0.36
19	Ige et al.	0.3	0.24, 0.36
20	Zetola et al	0.3	0.25, 0.36
21	O,connel et al	0.3	0.25, 0.36
22	Tola et al	0.3	0.25, 0.36
23	Ayana et al	0.3	0.25, 0.36
24	Tesfahugn et al	0.3	0.24, 0.36
25	Tesfaye et al	0.3	0.25, 0.36
26	Ambaw et al.	0.31	0.25, 0.36

### Publication Bias

**Fig. 5:**
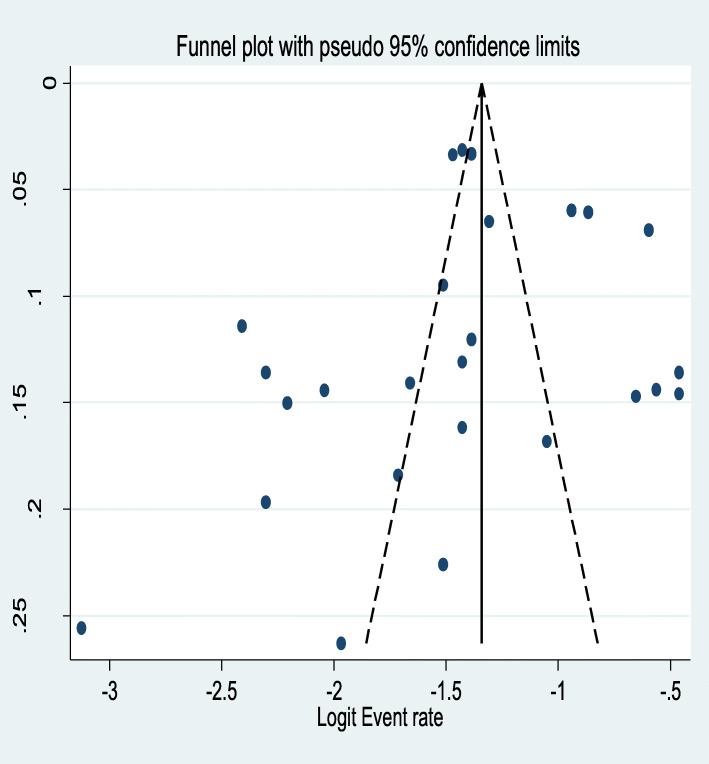
A funnel plot of logit event rate against its standard error

The Egger’s publication bias plot is near the origin and Egger’s tests *p*-value was (*P* = 0.58) showing the absence of publication bias for the prevalence of AUD among tuberculosis patients. This was also supported by asymmetrical distribution on the funnel plot for a logit event rate of prevalence of AUD in tuberculosis patients against its standard error **(**Fig. 5**).**

### Factors associated with alcohol use disorder among tuberculosis patients

Among 28 studies [11–13, 15, 17, 25, 33–53] included in the present meta-analysis, only eight [11–13, 25, 36, 37, 44, 48] reported the associated factors for alcohol use disorder among tuberculosis patients. Our qualitative synthesis for the socio-demographic factors associated with AUD in tuberculosis patients revealed that male gender [11, 12, 25, 36, 48], age older than 35 years [12], being single, divorced or widowed [12, 13], being unemployed [13], being black American [36], colored ethnicity [48], low level of education [12, 48], no educational background [11], low level of income (<70US$ per month) [12] and poverty [48]. Besides, being on category-II tuberculosis treatment(relapse and treatment failure) [12], TB retreatment patient status and non-adherence to anti-TB medication [48], patients with chronic/relapsing form of tuberculosis [37], patients with perceived TB stigma [11], patients who feel ashamed of having TB [11], people close to you would avoid you because of TB [11], HIV-co-infection and low HIV CD4-count [11], having cavitary lesions on chest radiographic examination [36], and smear-positive and culture-positive types of TB [36] were also the reported clinical and tuberculosis related factors for AUD (Table 4).

**Table 4:** Factors that increase the risk of alcohol use disorder in tuberculosis patients

Factors that increase the risk of AUD in tuberculosis patients	Factors that are protective of AUD in tuberculosis patients
❖ Socio-demographic factors Male gender [11, 12, 25, 36, 48] Age older than 35 years [12] Being single, divorced or widowed [12, 13] Being unemployed [13] Being black American [36] Coloured ethnicity [48] Low level of education [12, 48] No educational background [11] Low level of income (<70US$ per month) [12] and poverty [48]	❖ Socio-demographic factors 41 to 54 years of age [44] Higher educational achievement and marital relationship [12, 44] Female gender [12]
❖ Clinical and tuberculosis related factors Being on category-II tuberculosis treatment(relapse and treatment failure) [12] TB retreatment patient status and non-adherence to anti-TB medication [48] Patients with chronic/relapsing form of tuberculosis [37] Patients with perceived TB stigma [11] Patients who feel ashamed of having TB [11] People close to you would avoid you because of TB [11] HIV-co-infection and low HIV CD4-count [11] Having cavitary lesions on chest radiographic examination [36] Smear positive and culture positive types of TB [36]	❖ Clinical and tuberculosis related factors Tuberculosis treatment category I and III [12] Having extra pulmonary TB as compared to Pulmonary of mixed type of TB [36] Good tuberculosis medication adherence [48]
❖ Substance related variables Current tobacco use [44]	

Key: *HIV* Human Immune Virus, *TB* Tuberculosis

## Discussion

Alcohol use disorder in individuals with tuberculosis is an important driver for poor tuberculosis treatment outcomes [16]. In comparison to tuberculosis patients with no alcohol use disorder, those who have this problem are faced higher rates of treatment failure, relapse, and death. Despite this and other impacts that AUD poses on individuals with tuberculosis, to the knowledge of researchers of the present review; there is no aggregate evidence on the average prevalence of AUD among this target population. The present meta-analysis study, therefore, intended to narrow the gap in evidence in this area by supplementing solid evidence on the 1-year prevalence of alcohol use disorder and its associated factors in TB patients. The evidence obtained will be of paramount importance for public health practitioners and policymakers.

Therefore it was necessary to have an average estimate for the prevalence of AUD in the global context and the current meta-analysis was therefore rooted in this justification. The average prevalence of alcohol use disorder among tuberculosis patients using the random effect model was found to be 30% (95% CI: 24.00, 35.00). This result was consistent with the global average prevalence of AUD among individuals living with HIV/AIDS (29.80%) [57].

However, the present finding was higher when compared with the average prevalence of AUD in individuals living with HIV/AIDS in Africa (22%) [58]. It was also higher than the DSM-V 12 month prevalence of alcohol use disorder in the adult general population in the USA (13.9%) [59]. Moreover, the finding was higher than the average prevalence of AUD in the European, Australian, and Ethiopian general population in which the AUD prevalence was 11.1% [60], 11.8% [61], and 23.86% [62]. This could be the use of alcohol as a coping response for the psychological distress associated with the perceived severity of such life-threatening illness [60, 61].

On the contrary, the average prevalence of AUD in the present review was lower when compared with the prevalence of AUD in mental disorders (28 to 70%) [63]. Individuals with mental illness are most of the time in poor judgment and insight towards their illness which could be responsible for the higher prevalence of AUD.

The average 1-year prevalence of AUD in male participants as reported by a few of the studies was 33.6% and higher than the average prevalence of AUD in females (11.67%). This was consistent with earlier studies in Canada [64], the East African countries [65], and the United Kingdom [66]. The sociocultural expectations and influences between males and females could be responsible for this. Besides, differences in the neurochemistry of the brain between men and women like the higher release of dopamine in men than women with the same amount of alcohol intake could lead to the high level of AUD in men [67]. However, the exact justification for such differences is the recommendation for future researchers.

The average prevalence of AUD was with a slight heterogeneity (I^2^ = 57%, *p*-value < 0.001) from the difference between the 27 studies. For this reason, we did a sub-group analysis. Therefore we did a subgroup analysis and the average prevalence of AUD varied based on the continent of the review, the measurement tool for AUD, the type of study design, and the mean age of the participants.

The subgroup analysis based on the continent where the review was done showed a significant difference in the average prevalence of alcohol use disorder among tuberculosis patients. The average prevalence of AUD in tuberculosis patients was higher in Asia and Europe; 37% [11, 12, 17, 25, 35–38, 41–44, 47, 52] than the prevalence in US; 24% [11, 36] and Africa; 24% [13, 15, 33, 34, 39, 40, 45, 46, 48–51, 53]. This was supported by earlier studies [68]. Differences in the cultural context, variation in the availability of alcoholic drinks, and socio-economic variants could bring the variation. Furthermore, the difference in the number of articles included in the subgroup could also be responsible.

The average prevalence of AUD was 36% in studies that do not report the assessment tool for AUD [35, 36, 38, 40–43, 50] higher than the prevalence in studies that utilized AUDIT (26%) [11, 12, 15, 17, 25, 34, 44–49, 51–53]. This could happen due to the possibility of inclusion of mild levels of alcohol use and the overestimation of AUD in studies that did not report the measurement tool.

Besides, case control studies [15, 39, 41] provided higher prevalence of AUD (39%) than cross-sectional [12, 13, 17, 33, 34, 36, 37, 41, 43, 45, 49, 50](30%), cohort [25, 35, 38, 40, 44, 46, 48, 51–53](30%). The small number of studies included in the case control subgroup might affect the validity of the estimate and result in higher prevalence of AUD.

Finally, the mean age of the review participants included in the review was considered during the subgroup analysis and the average prevalence of AUD was 42% in studies with a mean age of the participants 40 years and above which is higher than the average prevalence of AUD in participants with a mean age of < 40 years (24%) and mean age not reported (27%). This was however in contradiction with the study finding by grant et al. [69] in which the prevalence of AUD declines over the age of 40 years.

Regarding the factors associated with AUD, our qualitative synthesis showed that the socio-demographic factors such as male gender [11, 12, 25, 36, 48], age older than 35 years [12], being single, divorced, or widowed [12, 13], being unemployed [13], being black American [36], colored ethnicity [48], low level of education [12, 48], no educational background [11], low level of income (< 70US$ per month) [12] and poverty [48] were related to AUD. Also, being on category-II tuberculosis treatment(relapse and treatment failure) [12], TB retreatment patient status and non-adherence to anti-TB medication [48], patients with chronic/relapsing form of tuberculosis [37], patients with perceived TB stigma [11], patients who feel ashamed of having TB [11], people close to you would avoid you because of TB [11], HIV-co-infection and low HIV CD4-count [11], having cavitary lesions on chest radiographic examination [36], and smear-positive and culture-positive types of TB [36] were also the reported clinical and tuberculosis related factors for AUD.

### Difference between included studies in the meta-analysis

Due to the slight heterogeneity in the present meta-analysis; we did a subgroup analysis. The result from subgroup analysis showed that the measurement tool employed to screen AUD, the continent where the study was done, the mean age of the participants studied, and type of the study design were identified as sources of difference between the 27 included studies. Furthermore, a single study leaves out analysis was done to screen studies outweighing the overall result but the average prevalence of AUD was not outweighed by a single particular study. This review is the first of its type to assess the average prevalence of alcohol use disorder in tuberculosis patients. The use of a pre-determined search strategy to obtain eligible articles minimizes the reviewer’s bias which increases the study quality. Besides, the implementation of subgroup analysis based on the measurement tool, the continent of the study, study setting, and mean age to identify the source of heterogeneity is also the strength of the present study. However, the use of a few studies in some groups of the subgroup analysis might affect the validity of estimate so that under or overestimation could occur. Moreover, the exclusion of articles published in non-English language might have also an effect on the magnitude of the average prevalence of AUD.

### Implications of this study for clinical practice, researchers, and policymakers

First, this review revealed that clinical practitioners who work in tuberculosis treatment centers have to be conscious that AUD is a common problem in tuberculosis patients and to offer patients management or treatment. Second, the high average estimated prevalence of AUD in tuberculosis patients obtained in the review as compared to the average estimated prevalence of AUD in the general population forces researchers to raise a question of why and what factors are responsible for this. Finally, the results inform policymakers and program planners that AUD is a significant public health concern for tuberculosis patients on treatment. This mitigates for a holistic approach during the clinical management of tuberculosis patients.

In conclusion, initiation of joint TB-alcohol collaborative activities including screening of all TB patients for alcohol use and screening of all patients consuming alcohol for TB should be initiated.

## Conclusion

The current review obtained a high average prevalence of AUD in tuberculosis patients and this prevalence varies with the measurement tool employed to screen AUD, the continent where the study was done, the mean age of the participants, and the type of the study design was. Moreover, the prevalence of AUD was higher in males than in females. Our qualitative synthesis showed that the factors such as male gender, older age, being single, being unemployed, low level of educational background, low level of income, Category-II tuberculosis treatment, TB retreatment patient, non-adherence to anti-TB medication, and perceived TB stigma was among also the associated factors for AUD in tuberculosis patients. Therefore early screening and management of AUD and its associated factors are essential in tuberculosis patients.

## Supplementary Information

**Additional file 1: Supplementary file 1.** A search strategy for the study.

## Data Availability

All data regarding this research work is included in the manuscript.

## Abbreviations

AUD: Alcohol Use Disorder
AUDIT: Alcohol Use Disorder Identification Test
ASSIST: Alcohol Smoking and Substance Involvement Screening Test
CC: Case-control
CS: Cross-sectional
DSM-IV: Diagnostic and Statistical Manual of Mental Disorders
DOT: Directly Observed Therapy
MINI: Mini-international Neuropsychiatric Interview
NA: Not Reported
PHCU: Primary Health Care Unit
PRISMA: Preferred Reporting Items for Systematic Reviews and Meta-analysis
RCT: Randomized Controlled Trial
SA: South Africa
US: United States
USA: United States of America
