# Supplementary material for: Prevalence and associated factors for alcohol use disorder among tuberculosis patients: a systematic review and meta-analysis study
Source: Subst Abuse Treat Prev Policy. 2021 Jan 3;16:2. doi: 10.1186/s13011-020-00335-w (PMC7778806; doi:10.1186/s13011-020-00335-w)
Supplement: Supplementary file 1 — Additional file 1: Supplementary file 1. A search strategy for the study. [file 13011_2020_335_MOESM1_ESM.docx]

**Search strategy**

1. **PubMed search:**  **(epidemiology OR Prevalence OR magnitude OR incidence) AND (Alcohol use OR alcohol abuse OR Alcohol dependence OR alcohol use disorder) AND (TB OR tuberculosis OR “MDR-TB” OR “multi-drug resistance tuberculosis”) AND (factor OR “associated factor” OR risk OR “risk factor” OR determinant)**.
2. **Embase search:** **Tuberculosis/exp OR Tuberculosis OR TB/exp OR TB OR MDR-TB/exp OR MDR-TB OR “Multi-drug-resistance TB”/exp OR "Multidrug-resistance" OR "alcohol use" OR "alcohol use"/exp OR “alcohol use disorder”/exp OR “alcohol use disorder” OR “alcohol abuse”/exp OR “alcohol abuse” OR “alcohol dependence”/exp OR “alcohol dependence”**
3. **Psych-Info search: (((alcohol use disorder.mp. [mp = title, abstract, heading word, table of contents, key concepts, original title, tests & measures]) OR (AUD.mp. [mp = title, abstract, heading word, table of contents, key concepts, original title, tests & measures]))) AND (((prevalence.mp. [mp = title, abstract, heading word, table of contents, key concepts, original title, tests & measures]) OR (magnitude.mp. [mp = title, abstract, heading word, table of contents, key concepts, original title, tests & measures] OR (epidemiology.mp. [mp = title, abstract, heading word, table of contents, key concepts, original title, tests & measures]))) AND (((tuberculosis.mp. [mp = title, abstract, heading word, table of contents, key concepts, original title, tests & measures]) OR (TB.mp. [mp = title, abstract, heading word, table of contents, key concepts, original title, tests & measures]))).**
